# Childhood Parental Emotions and Depression Among Middle-Aged and Elderly Chinese: The Mediating Role of Adverse Childhood School Neighborhood Friendship Experiences

**DOI:** 10.1155/da/3083436

**Published:** 2025-06-25

**Authors:** Gaoling Wang, Yuanxi Li, Yali Yu, Huiqing Tang, Ying Lu, Shaoliang Tang

**Affiliations:** School of Health Economics and Management, Nanjing University of Chinese Medicine, No. 138 Xianlin Avenue, Qixia District, Nanjing 210023, China

**Keywords:** adverse childhood school neighborhood friendship experiences, childhood parental emotions, depressive symptoms

## Abstract

**Background:** This study aimed to investigate the potential mediation effect of adverse childhood school neighborhood friendship experiences (ACSNFEs) in the relationship between childhood parental emotions and depressive symptoms.

**Methods:** The study extracted data from 9489 participants from the China Health and Retirement Longitudinal Study (CHARLS) of 2014 and 2020. Depressive symptoms were assessed by the 10-item Center for Epidemiological Studies Depression Scale (CES-D-10). Stepwise regression based on least squares regression models, bootstrap tests, and Karlson–Holm–Breen (KHB)-based logit regression models were applied to analyze.

**Results:** Negative childhood parental emotions (*β* = 0.2030 and *p* < 0.001), negative childhood mother's emotions (*β* = 0.3399 and *p* < 0.001), and negative childhood father's emotions (*β* = 0.3866 and *p* < 0.001) were all significantly associated with higher severity of depressive symptoms. Bootstrap tests showed that the proportion of ACSNFEs mediated for childhood parental emotions was 14.03%. For childhood mother's emotions and childhood father's emotions, the mediating proportions were 15.32% and 13.57%, respectively. Moreover, KHB tests showed that the mediating effect still existed.

**Conclusions:** The association between childhood parental emotions and depressive symptoms was partly mediated by ACSNFEs. Focus on developing parental emotional management ability, actively guiding parents to help children develop high-quality friendships, and promoting the development of psychological health.

## 1. Introduction

Depression is a worldwide health concern, with 5% of adults suffering from depression globally [1]. Individuals who suffer from depression are more likely than the average population to acquire physical illnesses like diabetes, cancer, and cardiovascular disease [2, 3]. According to the World Health Organization's Comprehensive Mental Health Action Plan 2013–2030, individuals with severe depression have a 40%–60% higher risk of premature death compared to the general population, and depression contributes to 4.3% of the global burden of disease, leading to significant socioeconomic implications [4]. Research indicates that depression significantly increases the risk of death from cardiovascular disease as well as from all other causes in Chinese people [5]. Psychotherapy can help improve dysfunction and reduce suicidal ideation in depressed elderly individuals [6]. Engaging in active research on the factors related to depression is paramount for augmenting the well-being of individuals, while simultaneously mitigating the psychological and financial burden imposed on families and society.

Life course experiences have an influential role in shaping disease risk trajectories [7]. Childhood is the beginning of individual life, and childhood experiences are significantly related to various health outcomes, such as psychological wellness, cognition, and chronic illnesses [8–10]. Cumulative advantage/disadvantage theory points out that risk exposures accumulate over time, and an individual's health and childhood experiences are intimately related [11, 12]. As an influential indicator of mental health, research on depression and childhood experiences has gradually become the focus of scholarly attention. A United Kingdom cohort study revealed that adverse childhood experiences (ACEs) in four dimensions: threat, family dysfunction, low parental relationships, and loss experiences are the main risk factors for the worsening of depression in later life [13]. ACEs are associated with an increased risk of depressive symptoms in a dose–response pattern [14]. Some scholars have explored that depressive symptoms mediate the relationship between ACEs and other disorders, while the pathways of action between ACEs and depressive symptoms remain to be further investigated [15, 16].

Family atmosphere and environment are crucial for physical and mental development, and adolescents living in an unwholesome family environment are prone to developing symptoms of anxiety and depression [17, 18]. Family dysfunction may have health consequences throughout life. Supportive family relationships and a safe and stable upbringing contribute to lifelong health [19], while childhood family economic disadvantage and family-related ACEs increase the risk of developing depressive symptoms and behavioral problems in later life [20]. Experiences of childhood family adversity such as parental abuse, poor parental relationships, and parental separation or divorce have long-term effects on mental health [21]. Adults who experienced emotional abuse or neglect in childhood had a 2.66–3.73 times higher likelihood of developing depression than those who did not suffer from childhood abuse [22]. Children of parents with mental illness (COPMI) may not only be prone to negative emotions like loneliness, shame, and insecurity [23], but also prefer to develop depression, substance abuse, schizophrenia, and physical disorders in adulthood [24]. ACEs-related research mainly focuses on parental behavior and family material life in childhood, such as parental abuse experiences, parenting styles, and childhood socioeconomic status [25–27]. More research is needed to investigate the relationship between childhood parental emotions and mental health.

Emotional expression emerges as a pivotal risk factor in the onset and progression of mental health disorders [28]. The degree of emotional expression within a family milieu has been found to prognosticate the likelihood of relapse among individuals diagnosed with depression and schizophrenia [29]. Expressed emotion theory posits that household environments characterized by heightened levels of expressed emotion contribute to exacerbating adolescent psychopathological distress [30]. The biobehavioral family model underscores a direct correlation between a passive family emotional climate and the manifestation of depressive symptoms in children [31]. This theoretical framework posits that the emotional tone set by family interactions plays a critical role in shaping the psychological well-being of offspring, particularly in the realm of depression vulnerability. A community-based study conducted in Finnish has revealed that children exhibit a heightened sensitivity to fluctuations in their parents' emotional states. Specifically, children who responded to low parental mood through overinvolvement or avoidance exhibited a greater prevalence of internalizing and externalizing symptoms including depression, in contrast to those who demonstrated active empathy or avoidance [32]. Early exposure to negative parental emotions may be one of the significant intergenerational transfers of depression developmental mechanism [33].

The social ecological models posit that an individual's health status is affected by multiple determinants, encompassing personal traits, interpersonal relationships, community neighborhoods, and public policies [34, 35]. The family serves as the foundational ecosystem for a child's life, and parental emotionally expressive behaviors have a far-reaching impact on children's emotional well-being and interpersonal interactions [36]. Social networks represent a momentous part of life activities, and positive social relationships can effectively enhance physical and mental health and longevity [37]. For children, social relationships mainly consist of family and friends. Campuses and community neighborhoods serve as the primary arenas where children cultivate and expand their peer relationships [38]. Parental vulnerability to moodiness or emotional instability may not only impede the development of social skills in their offspring but also be strongly associated with an increased likelihood of their children experiencing bullying victimization [39, 40]. Drawing on attachment theory, children who experience emotional abuse at the hands of their parents are more prone to developing insecure attachment styles, which in turn can precipitate a range of relationship difficulties and elevate the risk of being bullied or isolated [41]. Empirical research focusing on school-based bullying and victimization has demonstrated that children subjected to parental neglect and abuse are disproportionately likely to become targets of bullying [42, 43]. Adolescents suffering from family conflict tend to demonstrate reduced levels of collective efficacy and face an elevated risk of encountering neighborhood violence [44]. Scholars have researched the connection between childhood friendship and later depression. Positive early peer relationships are widely recognized to decrease depression symptoms in later life [45–47]. Research evidence also indicates that school bullying increases the possibility of developing mental disorders [48, 49]. There are intricate associations among childhood parental emotions, adverse childhood school neighborhood friendship experiences (ACSNFEs), and depressive symptoms.

**Figure 1:**
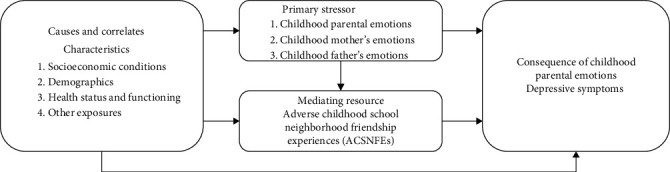
The proposed conceptual framework.

The stress process model provides a comprehensive framework for delving into the intricate relationships among diverse stressors from a theoretical perspective of stress and strain [50]. Drawing upon the extant literature and the stress process model, we have proposed a conceptual framework in Figure 1. We identify childhood parental emotions as the primary stressor that ultimately precipitates depressive symptoms (stress outcome). Furthermore, we posit that ACSNFEs not only constitute a secondary stressor contributing to depressive symptoms but also emerge as a consequence of childhood parental emotions, serving as a mediator between childhood parental emotions (primary stressor) and depressive symptoms (stress outcome). Existing research has focused primarily on the dose–response relationship between ACEs and depressive symptoms, and we have not found research on the impact of specific ACEs on adult mental health, such as childhood parental emotions and childhood friendships. Do childhood parental emotions influence depressive symptoms in middle-aged and older adults? Do ACSNFEs mediate between the two? The purpose of this study was to investigate the association between childhood parental emotions and depressive symptoms among middle-aged and elderly Chinese and whether ACSNFEs mediate the above relation.

## 2. Methods

### 2.1. Study Design and Participants

The data comes from the China Health and Retirement Longitudinal Study (CHARLS) 2014 life course survey and the 2020 national follow-up survey for cross-sectional analyses. As the first nationally representative tracking survey of China's middle-aged and older population, CHARLS provides multidimensional information on the basic situation, socioeconomic status, family, and health conditions of individuals aged 45 years and older in mainland China. High-quality microdata is valuable for scientific research on aging. The project has conducted multistage probability sample surveys in China since the initial investigation in 2011 and has completed four rounds of national tracking surveys in 2013, 2015, 2018, and 2020 [51]. CHARLS conducted a life course thematic survey in 2014 to collect data on respondents' family, education, and health experiences since birth. Furthermore, CHARLS obtained informed consent from each respondent and ethical approval from Peking University's Institutional Review Board (IRB00001052-11015) [52].

**Figure 2:**
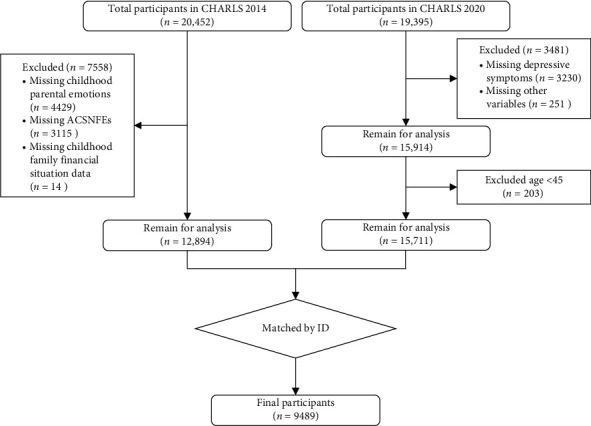
Sample selection process.

The study's target population was middle-aged and older adults aged 45 years and older. After excluding missing information, abnormal responses, and invalid data on key variables such as childhood parental emotions, ACSNFEs, and depressive symptoms, the final sample size was 9489. The sample screening process is shown in Figure 2.

### 2.2. Depressive Symptoms

Depressive symptoms were measured using the 10-item Center for Epidemiologic Studies Depression Scale (CES-D-10) set up in the CHARLS 2020 questionnaire. The CES-D-10 has been widely utilized to screen depressive symptoms among the Chinese population because of its reliability and validity. In this investigation, the internal consistency of the CES-D-10 scale based on CHARLS 2020 data demonstrated a reasonable level, with Cronbach' alpha value of 0.7927. Ten questions investigated the respondents' feelings and behaviors in the past week, and each question ranged from 0 to 3. Among the eight negative statements, 0 = rarely or not at all, 1 = little time, 2 = sometimes, and 3 = majority of the time. The positive statements “I felt hopeful about the future” and “I was happy” were given a reversal score, with 0 = majority of the time, 1 = sometimes, 2 = little time, and 3 = rarely or not at all. The scores for 10 items were eventually summed up, with a total depressive symptoms score ranging from 0 to 30, with higher scores reflecting more severe depressive symptoms.

### 2.3. Childhood Parental Emotions

Most scholars agree that emotions contain anger, sadness, joy, fear, and anxiety [53–55]. Combined with the design of the CHARLS life course questionnaire, childhood parental emotions in this study were comprehensively evaluated from three aspects. How often female and male guardians felt nervous and anxious, the frequency they got upset easily or felt panicky, and whether they had ever shown symptoms of sadness or depression that persisted for 2 weeks or longer (signs of sadness or depression mean low emotions rather than clinical depression). Responses to the questions “Did your female/male provider feel nervous and anxious when you were a child?” and “Did your female/male provider get upset easily or feel panicky when you were a child?”, 0 = rarely or never, 1 = some of the time, 2 = good part of the time, and 3 = most of the time. For the item “Did your female/male caregiver ever show symptoms of sadness or depression that lasted two weeks or more when you were a child?”, 0 = no and 1 = yes. Ultimately, the childhood parental emotions score is the sum of six scores, ranging from 0 to 14, with higher scores meaning more negative childhood parental emotions. The sum of three scores for the female guardian question was the childhood mother's emotions, ranging from 0 to 7, and the sum of the three scores for the male guardian question was the childhood father's emotions, ranging from 0 to 7.

### 2.4. ACSNFEs

ACSNFEs contained one question about adverse childhood neighborhood friendship experiences “How often did neighborhood kids bully you as a child?” and two questions about adverse childhood school friendship experiences “How often did school kids bully you as a child?”, “How often did you feel worried about your physical safety at school as a child?” through the CHARLS life course questionnaire. All entries were coded as 0 = never, 1 = rarely, 2 = sometimes, and 3 = often. Ultimately, the sum of the four question scores was the ACSNFEs score, ranging from 0 to 9. Higher scores correspond to more ACSNFEs.

### 2.5. Covariates

Based on existing research correlated with depression in middle-aged and elderly individuals, covariates included in this analysis were separated into three aspects: demographic characteristics, health status and behaviors, and socioeconomic conditions [56–58]. Demographic characteristics included age, gender, place of residence, education level, and marital status. The study found that the mental health of middle-aged and elderly Chinese is closely correlated to their place of residence. We reclassified residences into rural and urban according to the urban–rural division regulations of the National Bureau of Statistics of the People's Republic of China [59]. Since the exclusive of respondents with no educational experience, we reclassified education level into elementary school or below, middle school, and high school or above. According to the CHARLS questionnaire options, we described marital status in terms of the status of cohabitation with a spouse, with “living with no spouse” referring to those who are divorced, widowed, and have never been married, “living with no spouse temporarily” referring to those who do not live with their spouse due to work or other reasons, and “living with spouse” referring to those who are married and cohabiting with their spouse.

Health status and behaviors included self-reported health, smoking and drinking status, sleep duration, and chronic diseases. CHARLS listed 15 common chronic illnesses, such as asthma, diabetes, and heart diseases, and sequentially inquired about these illnesses from the respondents. We regarded those who self-reported having any chronic diseases as suffering from chronic diseases.

Socioeconomic conditions encompassed social participation, childhood family financial situation, household per capita expenditure, and medical insurance. Social participation helps mitigate loneliness and alleviate depression among older adults. There is a link between the interactions individuals have with others in society and depression, and research has shown that social participation helps mitigate loneliness and alleviate depression among older adults [58, 60]. We assessed social participation by determining whether the respondent had participated in eight social activities the previous month, including hanging out, playing mahjong, dancing, volunteering, etc. “No” means not participating in any of the eight activities, and “Yes” means participating in at least one of the eight activities in the past month. Through the question “How was your family's economic situation before you turned 17 compared to the average family in your community or village?” to assess childhood family financial status. We considered a little better or much better than them to be better than the average family, those who answered the same as them to be about the same as the average family, while those who answered a little worse or much worse than them as worse than the average household. As previous research has demonstrated that household consumption expenditure is more strongly connected with individual living standards than household income, we included household per capita expenditure as a proxy for the current economic status of the respondents [59]. At the same time, personal financial status is closely related to the accessibility of medical services, and medical insurance helps to improve the accessibility of medical services for those with lower socioeconomic status [61]. Medical insurance was a binary variable, with “Yes” and “No” representing whether or not to participate in medical insurance. Table S1 displays the coding of the relevant variables.

### 2.6. Statistical Analysis

Continuous variables were expressed as mean ± standard deviation, with intergroup differences assessed using *t*-tests. Categorical variables were presented as frequency distributions (proportions), and between-group comparisons were analyzed through chi-square tests. Baron and Kenny's stepwise regressions [62] were used to investigate the connection between childhood parental emotions, ACSNFEs, and depressive symptoms.

**Figure 3:**
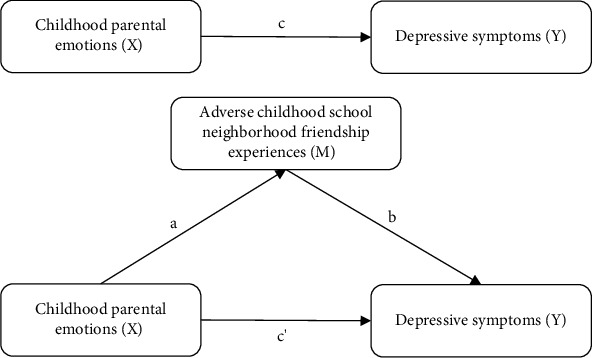
Research pathway hypothesis.

1. A least squares regression model was used to verify the association between childhood parental emotions and ACSNFEs (Figure 3, path a).
2. A least squares regression model was carried out between childhood parental emotions and depressive symptoms (Figure 3, path c).
3. With ACSNFEs incorporated, the relationship between childhood parental emotions and depressive symptoms was analyzed with ACSNFEs as a mediator variable (Figure 3, path c').

Meanwhile, nonparametric bootstrap tests were conducted with 5000 repeated samples to confirm the significance of the mediating effect. If the 95% confidence interval (CI) does not contain 0, the coefficient product is significant, indicating the presence of mediating effects. This study also utilized the KHB-based logit regression model for robustness testing. All statistical analyses were processed and analyzed using STATA 17.

## 3. Results

### 3.1. Description Statistics

From the characteristics of participants (Table 1), the 9489 participants consisted of 4401 females and 5088 males. Their average age was 59.90 (± 8.79) years, the average score for depressive symptoms was 8.15 (± 6.25), and the mean ACSNFEs score was 0.94 (± 1.51). In addition, the mean childhood parental emotions score of the 9489 samples in this study was 2.00 (± 3.06), and the mean childhood mother's emotions score was higher than that of fathers. Female participants demonstrated a significantly higher mean depressive symptom score (9.26 ± 6.43) compared to male counterparts. Conversely, male participants exhibited elevated scores in childhood parental emotions (2.06 ± 3.12) and ACSNFEs (0.99 ± 1.51), demonstrating statistically significant gender-based differences (*p* < 0.05). The male and female groups also differed significantly in sociodemographic characteristics, health status, and behavior (*p* < 0.05), whereas childhood mother's emotions and social participation showed no statistically significant variations.

### 3.2. Relevance Test

We constructed Model 1–Model 3 based on Baron and Kenny's stepwise regression method to investigate the relationship between childhood parental emotions, ACSNFEs, and depressive symptoms among middle-aged and elderly individuals (Table 2). In Model 1, after controlling covariates in three aspects, our analysis demonstrated a significant positive relationship between childhood parental emotions and ACSNFEs (*β* = 0.1123, SE = 0.0050, and *p* < 0.001), and children whose parents have negative emotions may have more negative friendship experiences in school and neighborhood. Model 2 showed a significant positive correlation between childhood parental emotions and depressive symptoms (*β* = 0.2361, SE = 0.0184, and *p* < 0.001), indicating that negative childhood parental emotions were significantly related to higher levels of depressive symptoms. In Model 3, the coefficients decreased with the addition of ACSNFEs, and there was still a significant positive association between childhood parental emotions and depressive symptoms (*β* = 0.2030, SE = 0.0189, and *p* < 0.001), indicating that ACSNFEs might have a partial mediating effect. At the same time, we also found that having chronic diseases was strongly associated with greater levels of depressive symptoms, whereas being male, living in an urban area, having a high level of education, being in better self-reported health, having a well-off childhood family, and participating in social activities and possessing medical insurance were strongly linked to reduced depressive symptoms.

In addition to the combined influence of childhood parental emotions on children, childhood mother's, and father's emotions may have different effects on children. The regression models showed that both childhood mother's emotions (*β* = 0.2046, SE = 0.0091, and *p* < 0.001) and childhood father's emotions (*β* = 0.1958, SE = 0.0101, and *p* < 0.001) were significantly and positively associated with ACSNFEs after accounting for relevant factors. In Model 2, depressive symptoms increased by 0.4014 and 0.4473 units for each unit increase in childhood mother's emotions (SE = 0.0333 and *p* < 0.001) and childhood father's emotions (SE = 0.0368 and *p* < 0.001). In Model 3, which incorporated ACSNFEs, depressive symptoms increased by 0.3399 and 0.3866 units for each unit increase in childhood mother's emotions (SE = 0.0341and *p* < 0.001) and childhood father's emotions (SE = 0.0374 and *p* < 0.001), respectively. In addition, compared to childhood parental emotions, the association between childhood family economic status and depressive symptoms increased significantly (Tables S2–S4).

**Figure 4:**
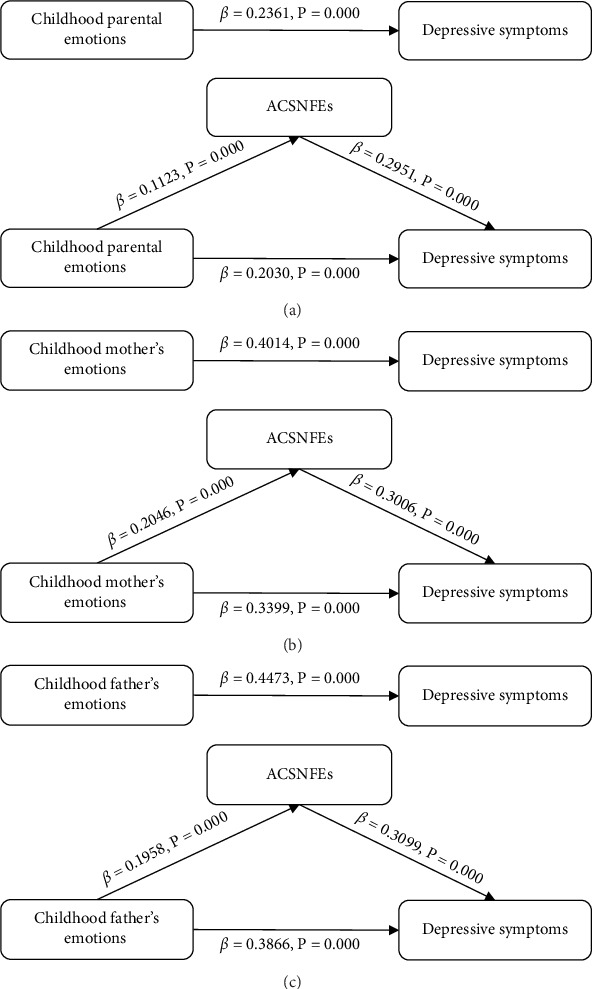
(A) ACSNFEs mediates the relationship between childhood parental emotions and depressive symptoms. (B) ACSNFEs mediates the relationship between childhood mother's emotions and depressive symptoms. (C) ACSNFEs mediates the relationship between childhood father's emotions and depressive symptoms.

Baron and Kenny's stepwise regression results were displayed as mediation pathway models illustrated in Figure 4. ACSNFEs partially mediate the relationship between childhood parental emotions and depressive symptoms.

### 3.3. Mediation Effect Analysis

The sampling number was 5000, the CI was 95%, the number of random seeds was 10,101, and bootstrap was applied to further validate the mediating effect of ACSNFEs (Table 3). The indirect effect of childhood parental emotions on depressive symptoms in middle-aged and older adults was 0.0331 (95% CI: 0.0236–0.0427), with a CI that did not include 0, indicating that the relationship between childhood parental emotions and depressive symptoms among middle-aged and elderly individuals was partially mediated by ACSNFEs, explaining 14.03% of the total effect. The indirect effect of childhood mother's emotions on depressive symptoms was 0.0615 (95% CI: 0.0441–0.0798), and the CI did not contain 0. The mediating effect of ACSNFEs on the relationship between childhood mother's emotions and depressive symptoms among middle-aged and elderly individuals was 15.32%. The indirect effect of childhood father's emotions on depressive symptoms was 0.0607 (95% CI: 0.0435–0.0779), with a CI not including 0. The mediating effect of ACSNFEs on childhood father's emotions on depressive symptoms among middle-aged and elderly individuals was 13.57%.

The findings of this study confirmed the impact pathway of childhood parental emotions on depressive symptoms, which is “childhood parental emotions-ACSNFEs- depressive symptoms.”

### 3.4. Robustness Test

We used logit regression for robustness testing based on the KHB approach for expanding linear model decomposability. Consistent with previous studies, we defined participants with CES-D-10 score equal to or higher than 10 as having depressive symptoms. Comparing the mean scores, ACSNFEs was recoded as dichotomous variables, with scores above and below the mean assigned a value of 1 and 0.

The results of the KHB test showed (Table 4) that after classifying the continuous variables and replacing the model, the indirect effect of childhood parental emotions on depressive symptoms was 0.0085 (95% CI: 0.0065–0.0117); the indirect effect of childhood mother's emotions on depressive symptoms was 0.0155 (95% CI: 0.0099–0.0211); childhood father's emotion on depressive symptoms was 0.0159 (95% CI: 0.0103–0.0216). All model regression coefficients were significantly at the 1% test level, and none of the 95% CIs contained 0, indicating that ACSNFEs play a partially mediating role between childhood parental emotions and depressive symptoms, proving the robustness of the models.

## 4. Discussion

Based on the 2014 and 2020 CHARLS data, we found a significant positive correlation between childhood parental emotions and depressive symptoms among middle-aged and elderly individuals. Meanwhile, ACSNFEs partially mediated the association.

Consistent with previous research, we found that negative childhood parental emotions were significantly associated with more severe depressive symptoms among middle-aged and elderly Chinese. A meta-analysis on childhood maltreatment and adult depression showed that adults who experienced childhood emotional abuse had an odds ratio (OR) of 3.73 for depression, and emotional abuse proved to be a significant trigger for subsequent episodes of depression [22]. By tracking depressive symptoms into middle and old age, we have further elucidated the long-term impact of parental negative emotions. Specific mechanisms regarding how childhood parental emotions affect later depressive symptoms remain unclear. Neurobiology offers one possible explanation. The development of posttraumatic stress disorder is associated with elevated levels of cortisol and norepinephrine, and the impact of early experiences on shaping the state of emotion regulation patterns and stress response mechanisms continues throughout life [63, 64]. Chaplin et al. examined parental emotional expression affecting neural responses in adolescents and showed that adolescents faced with negative parental expressions of emotion were more reactive in salient regions and had more severe symptoms of depression and anxiety [65]. Kennedy-Turner et al. [66] argue that negative family emotional expression leads to poorer mental health outcomes in adolescents since emotional expression might serve as a surrogate indicator for other dysfunctional family processes comparable to family stress. If children remain in an atypical emotional environment consisting of negative emotions for a long time, their emotional brain development, emotional processing ability development, and mental health growth will be seriously impeded [33, 67]. Chronic exposure to stressors can render individuals more vulnerable to stress and undermine their coping mechanisms, which in turn elevate the likelihood of mental illness development [68]. When dealing with patients with depressive symptoms, doctors can use childhood parental emotions as important assessment indicators and entry points for intervention, formulating more targeted treatment plans by recalling early life experiences.

The study found that ACSNFEs mediated the association between childhood parental emotions and depressive symptoms. Marquis-Brideau et al. [69] identified friendship quality as a mediator linking early family interactions to anxiety and depressive symptoms in adolescence, arguing that family alliances influence children's subsequent socioemotional functioning by shaping their ability to develop high-quality friendships. The results are consistent with our findings, and several potential mechanisms could explain it. First, parents will likely have some influence on later depression by affecting their children's interpersonal skills. Kaufman et al. [70] used spillover effects to reveal that poor parenting drives children to deteriorate peer relationships by causing social anxiety, and individuals who experience adverse childhood friendship experiences are more likely to experience depressive symptoms [71]. Second, negative self-perception may be the underlying pathway. Children who grow up in an atmosphere of negative parental emotions tend to be very insecure, and peer bullying accompanied by negative self-perceptions can reinforce an individual's low self-esteem and trigger depression [33, 72]. From the viewpoint of brain neural development, the development of the hippocampus is likely to be suppressed within a family environment characterized by negativity and indifference [73]. Childhood adverse friendship experiences not only damage the medial prefrontal cortex (PFC), a core neuron in the brain that regulates social behavior, but also increase the homozygous load and hypothalamic-pituitary-adrenal (HPA) axis activity, which in turn will jeopardize the brain function, behaviors, and mental health [74, 75]. Notably, positive interactions and mutual support with friends enhance brain sensitivity to social incentives [76], helping to buffer the risk of HPA axis dysregulation [77, 78]. In addition, it may be affected by intergenerational transmission. Parental emotions and behavioral patterns may also be passed on to their children, with long-term effects on their mental health development [79].

Using representative national sample survey data, this study focuses on the impact of childhood parental emotions as a specific childhood experience on depressive symptoms in middle-aged and older adults, bridging critical gaps in understanding how early-life socioemotional experiences shape late-life mental health, enriching the study of childhood experiences and mental health from the perspective of the life course. Life course theory points out that life events influence individual development and advocates confronting the complexity of social factors embedded in the evolution of the life course [7]. Childhood parental emotions reflect the characteristics of the micro-social structure of the family, and ACSNFEs reflect the meso-environments like school and community, which completes the theoretical framework of life course theory on the relationship between social structural factors and the development of individuals' mental health, providing a new research idea for the subsequent exploration of the interaction between early ecological environments and the development of individuals. By using ACSNFEs as mediating variables, the pathways between ACEs and depressive symptoms were explored in depth, providing a more nuanced mechanistic explanation for understanding the formation of depressive symptoms. The results of this research are also of great value in guiding mental health practice and policy formulation. Given the profound impact of childhood parental emotions and friendship experiences on individual mental health, relevant departments should introduce appropriate policies to strengthen mental health education for parents and social skills training for children, improving mental health screening mechanisms. Individuals with depression in middle-aged or older adults are encouraged to channel the impact of family emotional experiences by recalling positive childhood experiences, keeping emotional diaries, and seeking psychological counseling.

Nevertheless, this study still has some limitations. First, constrained by only the 2014 life course survey containing information about childhood experiences, this study used a cross-sectional design for the relationship between childhood parental emotions and ACSNFEs, precluding definitive causal inferences. Second, we used retrospective self-report questionnaires to measure core variables such as childhood parental emotions and ACSNFEs. It is imperative to acknowledge that, across the approximately three decades time interval within our research, recall, and information bias are inevitable, which could potentially impact our study. Third, we have not found a uniform index in existing studies to measure childhood parental emotions. The selection of indicators was mainly based on the existing literature and the items in the questionnaire that can reflect parental emotions were chosen. Although we controlled for a range of potential covariates as much as possible to ensure the robustness of our analytic results, there may still be confounding factors that we failed to account for adequately. In addition, we removed samples with missing values, and there may be limitations to the applicability of the results based on the 9489 sample.

Future research directions should prioritize longitudinal designs to elucidate causal pathways between childhood parental emotions and ACSNFEs. Furthermore, while our findings demonstrate partial mediation through ACSNFEs in childhood parental emotions and depressive symptoms, childhood parental emotions may also impact depressive symptoms through other mediating variables. Exploration and empirical validation of additional mediating variables are expected in subsequent investigations.

## 5. Conclusion

Our study uncovered a correlation between negative childhood parental emotions and higher levels of depressive symptoms among middle-aged and elderly Chinese individuals. Long-term exposure to negative parental emotions may lead to emotional, cognitive, and personality deviations in children, jeopardizing mental health in middle and late adulthood. Developing parental emotional management ability and helping them release negative emotions is paramount for children. At the same time, ACSNFEs have a partially mediating role. Actively guiding parents to help their children develop high-quality friendships, improving child interpersonal skills and resilience, and reducing childhood social isolation and bullying are crucial to promoting the development of psychological health.

## Figures and Tables

**Table 1:** Characteristics of participants.

Variable	Total (9489)	Female (4401)	Male (5088)	*p*
Depressive symptoms	8.15 ± 6.25	9.26 ± 6.45	7.19 ± 5.91	<0.001
Childhood parental emotions	2.00 ± 3.06	1.93 ± 2.98	2.06 ± 3.12	0.028
Childhood mother's emotions	1.13 ± 1.70	1.13 ± 1.70	1.14 ± 1.70	0.787
Childhood father's emotions	0.87 ± 1.52	0.80 ± 1.47	0.93 ± 1.56	<0.001
ACSNFEs	0.94 ± 1.51	0.88 ± 1.51	0.99 ± 1.51	<0.001
Age	59.90 ± 8.79	58.83 ± 8.65	60.83 ± 8.82	<0.001
Residence	—	—	—	<0.001
Rural	5945 (62.65)	2621 (59.55)	3324 (65.33)	—
Urban	3544 (37.35)	1780 (40.45)	1764 (34.67)	—
Education	—	—	—	<0.001
Elementary school or below	5194 (54.74)	2707 (61.51)	2487 (48.88)	—
Middle school	2776 (29.25)	1141 (25.93)	1635 (32.13)	—
High school or above	1519 (16.01)	553 (12.56)	966 (18.99)	—
Living with spouse or not	—	—	—	<0.001
Living with no spouse	1127 (11.88)	669 (15.20)	458 (9.00)	—
Living with no spouse temporarily	784 (8.26)	386 (8.77)	398 (7.82)	—
Living with spouse	7578 (79.86)	3346 (76.03)	4232 (83.18)	—
Self-reported health	—	—	—	<0.001
Very poor	573 (6.04)	293 (6.66)	280 (5.51)	—
Poor	1551 (16.35)	794 (18.04)	757 (14.88)	—
Fair	4950 (52.17)	2324 (52.80)	2626 (51.62)	—
Good	1226 (12.92)	524 (11.91)	701 (13.78)	—
Very good	1189 (12.53)	466 (10.59)	723 (14.21)	—
Smoke	—	—	—	<0.001
No	5421 (57.13)	4159 (94.50)	1262 (24.80)	—
Quit	1359 (14.32)	85 (1.93)	1274 (25.04)	—
Still have	2709 (28.55)	157 (3.57)	2552 (50.16)	—
Drink	—	—	—	<0.001
No	5608 (59.10)	3599 (81.78)	2009 (39.49)	—
Yes	3881 (40.90)	802 (18.22)	3079 (60.51)	—
Sleep time	1.77 ± 0.32	1.73 ± 0.34	1.81 ± 0.30	<0.001
Chronic	—	—	—	0.022
No	6075 (64.02)	2764 (62.80)	3311 (65.07)	—
Yes	3414 (35.98)	1637 (37.20)	1777 (34.93)	—
Social participation	—	—	—	0.191
No	4525 (47.69)	2067 (46.97)	2458 (48.31)	—
Yes	4964 (52.31)	2334 (53.03)	2630 (51.69)	—
Childhood family financial situation	—	—	—	<0.001
Worse	3345 (35.25)	1394 (31.67)	1951 (38.34)	—
Average	5128 (54.04)	2438 (55.40)	2690 (52.87)	—
Better	1016 (10.71)	569 (12.93)	447 (8.79)	—
Medical insurance	—	—	—	0.012
No	327 (3.45)	174 (3.95)	153 (3.01)	—
Yes	9162 (96.55)	4227 (96.05)	4935 (96.99)	—
Household per capita expenditure	6.37 ± 0.97	6.41 ± 0.96	6.34 ± 0.97	0.001

*Note:* Proportions in parentheses.

Abbreviation: ACSNFEs, adverse childhood school neighborhood friendship experiences.

**Table 2:** Regression results for childhood parental emotions and its items.

Variable	Model 1 ACSNFEs			Model 2 depressive symptoms			Model 3 depressive symptoms		
	*β* (SE)	95% CI	*p*	*β* (SE)	95% CI	*p*	*β* (SE)	95% CI	*p*
Childhood parental emotions	0.1123 (0.0050)	[0.102, 0.122]	<0.001	0.2361 (0.0184)	[0.200, 0.272]	<0.001	0.2030 (0.0189)	[0.166, 0.240]	<0.001
Childhood mother's emotions	0.2046 (0.0091)	[0.187, 0.222]	<0.001	0.4014 (0.0333)	[0.336, 0.467]	<0.001	0.3399 (0.0341)	[0.273, 0.407]	<0.001
Childhood father's emotions	0.1958 (0.0101)	[0.176, 0.216]	<0.001	0.4473 (0.0368)	[0.375, 0.519]	<0.001	0.3866 (0.0374)	[0.313, 0.460]	<0.001

*Note:* All control variables were included in the model.

Abbreviations: ACSNFEs, adverse childhood school neighborhood friendship experiences; CI, confidence interval; SE, standard error.

**Table 3:** Bootstrap examination of ACSNFEs.

Effect	*β*	Bootstrap SE	*p*	95% CI		Mediation (%)
				Lower	Upper	
Childhood parental emotions–ACSNFEs–depressive symptoms						
Indirect effect	0.0331	0.0049	0.000	0.0236	0.0427	14.03
Direct effect	0.2030	0.0205	0.000	0.1629	0.2431	—
Total effect	0.2361	0.0202	0.000	0.1964	0.2758	—
Childhood mother's emotions–ACSNFEs–depressive symptoms						
Indirect effect	0.0615	0.0089	0.000	0.0441	0.0789	15.32
Direct effect	0.3399	0.0368	0.000	0.2678	0.4120	—
Total effect	0.4014	0.0365	0.000	0.3299	0.4729	—
Childhood father's emotions–ACSNFEs–depressive symptoms						
Indirect effect	0.0607	0.0088	0.000	0.0435	0.0779	13.57
Direct effect	0.3866	0.0407	0.000	0.3068	0.4664	—
Total effect	0.4473	0.0404	0.000	0.3681	0.5265	—

Abbreviations: ACSNFEs, adverse childhood school neighborhood friendship experiences; CI, confidence interval; SE, standard error.

**Table 4:** KHB test of ACSNFEs.

Effect	*β*	Bootstrap SE	*p*	95% CI		Mediation (%)
				Lower	Upper	
Childhood parental emotions–ACSNFEs–depressive symptoms						
Indirect effect	0.0085	0.0016	0.000	0.0065	0.0117	10.61
Direct effect	0.0719	0.0081	0.000	0.0560	0.0878	—
Total effect	0.0805	0.0080	0.000	0.0648	0.0961	—
Childhood mother's emotions–ACSNFEs–depressive symptoms						
Indirect effect	0.0155	0.0028	0.000	0.0099	0.0211	11.16
Direct effect	0.1233	0.0147	0.000	0.0946	0.1521	—
Total effect	0.1388	0.0144	0.000	0.1105	0.1672	—
Childhood father's emotions–ACSNFEs–depressive symptoms						
Indirect effect	0.0159	0.0029	0.000	0.0103	0.0216	10.62
Direct effect	0.1341	0.0161	0.000	0.1026	0.1656	—
Total effect	0.1501	0.0159	0.000	0.1189	0.1812	—

Abbreviations: ACSNFEs, adverse childhood school neighborhood friendship experiences; CI, confidence interval; SE, standard error.

## Data Availability

All the data are publicly available at https://charls.pku.edu.cn/.

## Nomenclature

ACSNFEs: Adverse childhood school neighborhood friendship experiences
ACEs: Adverse childhood experiences
CHARLS: China Health and Retirement Longitudinal Study
CES-D-10: 10-item Center for Epidemiological Studies Depression Scale
COPMI: Children of parents with mental illness
HPA: Hypothalamic-pituitary-adrenal
KHB: Karlson–Holm–Breen
OR: Odds ratio.

## Supporting Information

Supporting Information Table S1: Coding of variables. We display the coding of variables used in this study. Table S2: Regression results for childhood parental emotions, ACSNFEs, and depression. The detailed regression results for childhood parental emotions, ACSNFEs, and depression. Table S3: Regression results for childhood mother's emotions, ACSNFEs, and depression. The detailed regression results for childhood mother's emotions, ACSNFEs, and depression. Table S4: Regression results for childhood father's emotions, ACSNFEs, and depression. The detailed regression results for childhood father's emotions, ACSNFEs, and depression.
