# Supplementary material for: Childhood Parental Emotions and Depression Among Middle-Aged and Elderly Chinese: The Mediating Role of Adverse Childhood School Neighborhood Friendship Experiences
Source: Depress Anxiety. 2025 Jun 25;2025:3083436. doi: 10.1155/da/3083436 (PMC12221546; doi:10.1155/da/3083436)
Supplement: Supporting Information — Table S1: Coding of variables. We display the coding of variables used in this study. Table S2: Regression results for childhood parental emotions, ACSNFEs, and depression. The detailed regression results for childhood parental emotions, ACSNFEs, and depression. Table S3: Regression results for childhood mother's emotions, ACSNFEs, and depression. The detailed regression results for childhood mother's emotions, ACSNFEs, and depression. Table S4: Regression results for childhood father's emotions, ACSNFEs, and depression. The detailed regression results for childhood father's emotions, ACSNFEs, and depression. [file 3083436.f1.doc]

**Appendix**

**Table S1.** Coding of variables

| **Variable** | **Coding** |
| --- | --- |
| Depression | 0~30 |
| Childhood parental emotions | 0~14 |
| Childhood mother’s emotions | 0~7 |
| Childhood father’s emotions | 0~7 |
| ACSNFEs | 0~9 |
| Age | ≥45 |
| Gender | Female=0, Male=1 |
| Residence | Rural=0, Urban=1 |
| Education | Elementary School or below=1, Middle School=2, High School or above =3 |
| Living with spouse or not | Living with no spouse=0, Living with no spouse temporarily=1, Living with spouse=2 |
| Self-reported health | Very poor=1, Poor=2, Fair=3, Good=4, Very good=5 |
| Smoke | No=0, Quit=1, Still have=2 |
| Drink | No=0, Yes=1 |
| Sleep time | Take the log of sleep time |
| Chronic | No=0, Yes=1 |
| Social participation | No=0, Yes=1 |
| Childhood family financial situation | Worse=1, Average=2, Better=3 |
| Medical insurance | No=0, Yes=1 |
| Household per capita expenditure | Take the log of household per capita expenditure |

**Table S2. Regression results for childhood parental emotions, ACSNFEs,** and depression

| **Variable** | **Model 1** | **Model 2** | **Model 3** |
| --- | --- | --- | --- |
|  | **ACSNFEs** | **Depression** | **Depression** |
| **Childhood parental emotions** | 0.1123^***^ | 0.2361^***^ | 0.2030^***^ |
|  | (0.0050) | (0.0184) | (0.0189) |
| **ACSNFEs** |  |  | 0.2951^***^ |
|  |  |  | (0.0376) |
| **Age** | -0.0234^***^ | 0.0036 | 0.0105 |
|  | (0.0018) | (0.0068) | (0.0068) |
| **Gender (Male)** | 0.1834^***^ | -1.3204^***^ | -1.3745^***^ |
|  | (0.0448) | (0.1646) | (0.1643) |
| **Residence (Urban)** | -0.0139 | -0.8255^***^ | -0.8214^***^ |
|  | (0.0332) | (0.1218) | (0.1215) |
| **Education** |  |  |  |
| Middle School | -0.0610^*^ | -0.6728^***^ | -0.6548^***^ |
|  | (0.0353) | (0.1295) | (0.1291) |
| High School or above | -0.0341 | -1.1222^***^ | -1.1122^***^ |
|  | (0.0458) | (0.1680) | (0.1675) |
| **Living with spouse or not** |  |  |  |
| Living with no spouse temporarily | 0.0697 | -0.3118 | -0.3324 |
|  | (0.0698) | (0.2564) | (0.2556) |
| Living with spouse | -0.0601 | -1.4371^***^ | -1.4194^***^ |
|  | (0.0480) | (0.1762) | (0.1756) |
| **Self-reported health** |  |  |  |
| Poor | 0.0362 | -2.6828^***^ | -2.6935^***^ |
|  | (0.0706) | (0.2592) | (0.2583) |
| Fair | -0.0164 | -5.2552^***^ | -5.2503^***^ |
|  | (0.0650) | (0.2385) | (0.2378) |
| Good | -0.0564 | -7.3023^***^ | -7.2857^***^ |
|  | (0.0753) | (0.2765) | (0.2757) |
| Very good | -0.1873^**^ | -7.8121^***^ | -7.7569^***^ |
|  | (0.0759) | (0.2787) | (0.2779) |
| **Smoke** |  |  |  |
| Quit | -0.0510 | -0.0583 | -0.0433 |
|  | (0.0531) | (0.1951) | (0.1945) |
| Still have | -0.0373 | 0.0952 | 0.1062 |
|  | (0.0453) | (0.1665) | (0.1660) |
| **Drink (Yes)** | 0.0258 | -0.3889^***^ | -0.3966^***^ |
|  | (0.0341) | (0.1251) | (0.1247) |
| **Sleep time** | -0.1583^***^ | -3.6155^***^ | -3.5688^***^ |
|  | (0.0478) | (0.1755) | (0.1751) |
| **Chronic (Yes)** | 0.0769^**^ | 0.4090^***^ | 0.3863^***^ |
|  | (0.0318) | (0.1169) | (0.1165) |
| **Social participation (Yes)** | 0.0549^*^ | -0.3825^***^ | -0.3987^***^ |
|  | (0.0302) | (0.1109) | (0.1106) |
| **Childhood family financial situation** |  |  |  |
| Average | -0.2626^***^ | -0.4371^***^ | -0.3596^***^ |
|  | (0.0330) | (0.1211) | (0.1212) |
| Better | -0.2211^***^ | -0.6114^***^ | -0.5461^***^ |
|  | (0.0534) | (0.1961) | (0.1957) |
| **Medical insurance (Yes)** | -0.0849 | -0.7030^**^ | -0.6779^**^ |
|  | (0.0815) | (0.2990) | (0.2981) |
| **Household per capita expenditure** | -0.0004 | -0.4128^***^ | -0.4127^***^ |
|  | (0.0166) | (0.0610) | (0.0609) |
| **_cons** | 2.6036^***^ | 25.3375^***^ | 24.5692^***^ |
|  | (0.2136) | (0.7841) | (0.7877) |
| ***N*** | 9489 | 9489 | 9489 |
| **adj. *R*^2^** | 0.092 | 0.285 | 0.289 |

Note: Standard errors in parentheses; ^*^ *p* < 0.1, ^**^ *p* < 0.05, ^***^ *p* < 0.01.

**Table S3. Regression results for childhood mother’s emotions, ACSNFEs,** and depression

| **Variable** | **Model 4** | **Model 5** | **Model 6** |
| --- | --- | --- | --- |
|  | **ACSNFEs** | **Depression** | **Depression** |
| **Childhood mother’s emotions** | 0.2046^***^ | 0.4014^***^ | 0.3399^***^ |
|  | (0.0091) | (0.0333) | (0.0341) |
| **ACSNFEs** |  |  | 0.3006^***^ |
|  |  |  | (0.0377) |
| **Age** | -0.0237^***^ | 0.0031 | 0.0102 |
|  | (0.0018) | (0.0068) | (0.0068) |
| **Gender (Male)** | 0.1978^***^ | -1.2903^***^ | -1.3498^***^ |
|  | (0.0448) | (0.1648) | (0.1644) |
| **Residence (Urban)** | -0.0198 | -0.8394^***^ | -0.8334^***^ |
|  | (0.0332) | (0.1219) | (0.1215) |
| **Education** |  |  |  |
| Middle School | -0.0633^*^ | -0.6831^***^ | -0.6640^***^ |
|  | (0.0352) | (0.1296) | (0.1292) |
| High School or above | -0.0332 | -1.1265^***^ | -1.1165^***^ |
|  | (0.0457) | (0.1682) | (0.1676) |
| **Living with spouse or not** |  |  |  |
| Living with no spouse temporarily | 0.0620 | -0.3242 | -0.3428 |
|  | (0.0698) | (0.2567) | (0.2559) |
| Living with spouse | -0.0593 | -1.4386^***^ | -1.4208^***^ |
|  | (0.0480) | (0.1764) | (0.1758) |
| **Self-reported health** |  |  |  |
| Poor | 0.0397 | -2.6768^***^ | -2.6888^***^ |
|  | (0.0705) | (0.2594) | (0.2586) |
| Fair | -0.0091 | -5.2486^***^ | -5.2458^***^ |
|  | (0.0649) | (0.2388) | (0.2380) |
| Good | -0.0456 | -7.2900^***^ | -7.2763^***^ |
|  | (0.0753) | (0.2769) | (0.2760) |
| Very good | -0.1785^**^ | -7.8066^***^ | -7.7529^***^ |
|  | (0.0759) | (0.2791) | (0.2782) |
| **Smoke** |  |  |  |
| Quit | -0.0481 | -0.0532 | -0.0387 |
|  | (0.0531) | (0.1953) | (0.1946) |
| Still have | -0.0359 | 0.1002 | 0.1110 |
|  | (0.0453) | (0.1666) | (0.1661) |
| **Drink (Yes)** | 0.0209 | -0.3986^***^ | -0.4049^***^ |
|  | (0.0341) | (0.1252) | (0.1248) |
| **Sleep time** | -0.1587^***^ | -3.6262^***^ | -3.5785^***^ |
|  | (0.0478) | (0.1757) | (0.1752) |
| **Chronic (Yes)** | 0.0793^**^ | 0.4165^***^ | 0.3926^***^ |
|  | (0.0318) | (0.1170) | (0.1166) |
| **Social participation (Yes)** | 0.0468 | -0.3966^***^ | -0.4107^***^ |
|  | (0.0302) | (0.1110) | (0.1107) |
| **Childhood family financial situation** |  |  |  |
| Average | -0.2583^***^ | -0.4495^***^ | -0.3718^***^ |
|  | (0.0330) | (0.1213) | (0.1213) |
| Better | -0.2179^***^ | -0.6257^***^ | -0.5602^***^ |
|  | (0.0534) | (0.1963) | (0.1958) |
| **Medical insurance (Yes)** | -0.0820 | -0.6947^**^ | -0.6701^**^ |
|  | (0.0814) | (0.2993) | (0.2984) |
| **PCE** | -0.0005 | -0.4142^***^ | -0.4140^***^ |
|  | (0.0166) | (0.0611) | (0.0609) |
| **_cons** | 2.5996^***^ | 25.4075^***^ | 24.6261^***^ |
|  | (0.2134) | (0.7848) | (0.7883) |
| ***N*** | 9489 | 9489 | 9489 |
| **adj. *R*^2^** | 0.093 | 0.283 | 0.288 |

Note: Standard errors in parentheses; ^*^ *p* < 0.1, ^**^ *p* < 0.05, ^***^ *p* < 0.01.

**Table S4. Regression results for childhood father’s emotions, ACSNFEs,** and depression

| **Variable** | **Model7** | **Model 8** | **Model 9** |
| --- | --- | --- | --- |
|  | **ACSNFEs** | **Depression** | **Depression** |
| **Childhood father’s emotions** | 0.1958^***^ | 0.4473^***^ | 0.3866^***^ |
|  | (0.0101) | (0.0368) | (0.0374) |
| **ACSNFEs** |  |  | 0.3099^***^ |
|  |  |  | (0.0374) |
| **Age** | -0.0232^***^ | 0.0041 | 0.0113^*^ |
|  | (0.0019) | (0.0068) | (0.0068) |
| **Gender (Male)** | 0.1725^***^ | -1.3478^***^ | -1.4013^***^ |
|  | (0.0451) | (0.1648) | (0.1644) |
| **Residence (Urban)** | -0.0118 | -0.8177^***^ | -0.8140^***^ |
|  | (0.0334) | (0.1220) | (0.1215) |
| **Education** |  |  |  |
| Middle School | -0.0674^*^ | -0.6801^***^ | -0.6592^***^ |
|  | (0.0355) | (0.1296) | (0.1292) |
| High School or above | -0.0437 | -1.1367^***^ | -1.1231^***^ |
|  | (0.0460) | (0.1681) | (0.1675) |
| **Living with spouse or not** |  |  |  |
| Living with no spouse temporarily | 0.0810 | -0.2894 | -0.3145 |
|  | (0.0703) | (0.2566) | (0.2557) |
| Living with spouse | -0.0655 | -1.4454^***^ | -1.4251^***^ |
|  | (0.0483) | (0.1763) | (0.1757) |
| **Self-reported health** |  |  |  |
| Poor | 0.0315 | -2.6924^***^ | -2.7021^***^ |
|  | (0.0710) | (0.2594) | (0.2585) |
| Fair | -0.0351 | -5.2880^***^ | -5.2771^***^ |
|  | (0.0653) | (0.2386) | (0.2378) |
| Good | -0.0801 | -7.3451^***^ | -7.3203^***^ |
|  | (0.0758) | (0.2767) | (0.2757) |
| Very good | -0.2134^***^ | -7.8566^***^ | -7.7905^***^ |
|  | (0.0764) | (0.2788) | (0.2780) |
| **Smoke** |  |  |  |
| Quit | -0.0544 | -0.0655 | -0.0486 |
|  | (0.0535) | (0.1953) | (0.1946) |
| Still have | -0.0350 | 0.0974 | 0.1082 |
|  | (0.0456) | (0.1666) | (0.1660) |
| **Drink (Yes)** | 0.0308 | -0.3778^***^ | -0.3874^***^ |
|  | (0.0343) | (0.1252) | (0.1248) |
| **Sleep time** | -0.1727^***^ | -3.6357^***^ | -3.5822^***^ |
|  | (0.0481) | (0.1756) | (0.1751) |
| **Chronic (Yes)** | 0.0790^**^ | 0.4101^***^ | 0.3856^***^ |
|  | (0.0320) | (0.1170) | (0.1166) |
| **Social participation (Yes)** | 0.0650^**^ | -0.3613^***^ | -0.3815^***^ |
|  | (0.0304) | (0.1110) | (0.1106) |
| **Childhood family financial situation** |  |  |  |
| Average | -0.2980^***^ | -0.4913^***^ | -0.3990^***^ |
|  | (0.0330) | (0.1206) | (0.1207) |
| Better | -0.2548^***^ | -0.6623^***^ | -0.5833^***^ |
|  | (0.0536) | (0.1959) | (0.1955) |
| **Medical insurance (Yes)** | -0.0838 | -0.7038^**^ | -0.6778^**^ |
|  | (0.0820) | (0.2993) | (0.2983) |
| **PCE** | -0.0020 | -0.4151^***^ | -0.4145^***^ |
|  | (0.0167) | (0.0611) | (0.0609) |
| **_cons** | 2.7243^***^ | 25.5138^***^ | 24.6694^***^ |
|  | (0.2146) | (0.7837) | (0.7875) |
| ***N*** | 9489 | 9489 | 9489 |
| **adj. *R*^2^** | 0.081 | 0.284 | 0.289 |

Note: Standard errors in parentheses; ^*^ *p* < 0.1, ^**^ *p* < 0.05, ^***^ *p* < 0.01.
